# Revising the Role of Integrin Subunit β4 Expression in Colon Cancer Progression and Survival

**DOI:** 10.1007/s12029-021-00787-8

**Published:** 2022-02-03

**Authors:** Eva Rademaker, Esther Bastiaannet, Jan Oosting, Neeltje G. Dekker-Ensink, Peter J. K. Kuppen, Noel F. C. C. de Miranda, Gerrit J. Liefers

**Affiliations:** 1 Department of Surgical Oncology, Leiden University Medical Center, P.O. Box 9600, Leiden, 2300 RC The Netherlands; 2 Department of Pathology, Leiden University Medical Center, Leiden, The Netherlands

**Keywords:** Integrin β4, β4 subunit, Biomarker, Colon cancer, Invasion, Hemidesmosome

## Abstract

**Purpose:**

Integrin subunit β4 (β4) has been proposed to play an important role in colon cancer progression through its involvement in hemidesmosome disassembly processes and tumor cell migration. However, the association between β4 expression and clinicopathological outcomes in colon cancer remains unclear.

**Methods:**

Expression of β4 was assessed by immunohistochemistry in a large cohort of 651 colon cancer patients, the largest colon cancer cohort so far. Chi-squared tests were used to study the association between β4 expression and clinicopathological features. Overall and disease-free survival were assessed by Cox proportional hazard models.

**Results:**

Loss of β4 expression was associated with local tumor invasion. Only 17.9% of the pT1 tumors displayed weak β4 expression level versus 28.1% of pT4 tumors, and 25.0% of the pT1 tumors had a high expression level versus 8.6% of the pT4 tumors (*p* = 0.012). No association between β4 expression and overall (*p* = 0.845) or disease-free survival (*p* = 0.767) was encountered, which disputes the role of β4 as a biomarker of malignant behavior in colon cancer.

**Conclusion:**

Contradictory reports have suggested opposite roles for β4 expression in (colon) cancer progression. In the present large cohort of colon cancer patients, we found that β4 expression was not associated with worse clinical prognosis, but decreased with advanced pathological tumor stage. Future studies should establish whether loss of β4 expression promotes invasive characteristics of colon cancer cells.

## Background

Integrins are transmembrane receptors consisting of two subunits (α and β) that bind to extracellular matrix (ECM) components. Integrin α6β4 is commonly expressed at the basal surface of intestinal epithelial cells where it interacts with its ligand laminin, at the basement membrane [1]. The integrin β4 subunit (β4) has exceptional characteristics compared to other integrin subunits, because of its long cytoplasmic tail containing 1017 amino acids that interacts directly with the intermediate filament plectin and thereby the cell’s cytoskeleton [2–4]. This association forms a stable adhesive structure denominated hemidesmosome.

Acquisition of migratory and invasive properties by cancer cells is a hallmark of carcinogenesis and the loss of hemidesmosome-mediated adhesion can be an important enabler [3]. Several signaling pathways can affect hemidesmosome stability, namely, through phosphorylation of the cytoplasmic tail of β4 [5–7]. As a consequence, the hemidesmosome structure is disrupted and α6β4 is mobilized from this complex, thereby enabling β4 to trigger the activation of the PI3K and Ras-MAPK signaling pathways [8, 9]. In turn, their activation drives a number of oncogenic features in cancer cells including proliferation, migration, and resistance to apoptosis [10].

Previous studies have shown that higher expression of β4 is associated with malignant behavior and poor clinical prognosis in bladder cancer [11], cervical cancer with a squamous histology [12], squamous cell carcinomas of the head and neck [13], non-small cell lung cancer of squamous subtype [14], pancreatic cancer [15], thyroid cancer [16], and basal-like breast cancer [17]. Furthermore, α6β4 deficiency was demonstrated to lead to higher apoptotic rates, in vivo, of human breast carcinoma cells, thereby confirming the close link between this integrin and cell survival processes [18].

However, no consensus has yet been achieved regarding the potential role of β4 in colorectal cancer progression. Several studies proposed that β4 is upregulated in carcinomas in comparison with normal mucosa or adenomas while others have found no relevant associations, or even diminished expression of β4 in invasive lesions [19–25]. Mishra and colleagues demonstrated that circulating tumor cells, as expected, had less adhesive properties, but also lower β4 expression as compared to the primary tumor [26].

It is expected that the cellular localization of this integrin also has profound impact on cell behavior as a basal, membranous localization is typical of normal colonic epithelium while diffuse β4 expression might correspond to an oncogenic pattern [27, 28]. Accordingly, the expression levels and localization of β4 have been associated to cellular differentiation: membranous expression of β4 was shown to be increased in well and moderately differentiated carcinomas, whereas largely absent in poorly differentiated tumors [22, 24, 29]. On the other hand, increased β4 expression but with a diffuse localization has also been reported in poorly differentiated tumors [21]. For several cancer types, a poorly differentiated histology associates with more aggressive behavior, which is somewhat contradictory to the proposed oncogenic role of β4 overexpression in some studies [30]. This apparent contradiction, together with the distinct roles for β4 according to its cellular localization and distinct isoforms of both the α6 and β4 subunit, may partly explain the conflicting reports on this integrin [22, 31]. When looking into clinical parameters, specifically in colorectal cancer, the association between clinical outcomes and level of β4 expression is also debatable: whereas one study did not find any association between β4 expression and tumor stage, another study reported that β4 expression levels positively correlated with clinical stage [21, 23].

To elucidate the relation between the expression of this integrin subunit β4 and both clinical outcome and malignant features of the colon tumor, we evaluated its expression in tissues derived from 653 colon cancer patients.

## Methods

### Patient Cohort

The patient cohort was previously described by Reimers and colleagues [32]. Patient data was anonymized in line with the national ethics guidelines (Code for Proper Secondary Use of Human Tissue, Dutch Federation of Medical Scientific Societies) and the analysis was done according to the code of conduct for responsible use. In brief, it comprises 1026 patients diagnosed with a colon tumor at time of diagnosis and who underwent resection between 2002 and 2008. Tissue microarrays (TMAs) were constructed with TMA Master (3DHistech Ltd). They were comprised of three 1.0-mm diameter cores, extracted from archival formalin-fixed paraffin-embedded tumor tissues under supervision of a pathologist. After immunohistochemical analysis, 653 patients were determined to be represented by good-quality, evaluable TMA cores.

### Immunohistochemistry

Four-micrometer TMA sections were deparaffinized in xylene for three consecutive steps of 15 min after which slides were washed in decreasing concentrations of ethanol (100%-70%-50%). Endogenous peroxidase activity was blocked by using a solution of 0.3% hydrogen peroxide diluted in methanol (Merck Millipore, Burlington, MA, USA) for 20 min. Heat-induced antigen retrieval was performed in boiling citrate buffer (10 mM, pH 6.0) for 10 min in a microwave. After washing with PBS-Tween (0.2%), all slides were incubated overnight with anti-integrin β4 rabbit monoclonal antibodies, diluted 1:400 in PBA-BSA (1%) (D8P6C, Cell Signaling, USA). The next day, slides were washed three times in PBS-Tween and incubated for 1 h with poly-horseradish peroxidase solution (Immunologic, The Netherlands). DAB + chromogen (DAKO, Agilent Technologies, USA) was used for chromogenic development and counterstaining was done with hematoxylin (Thermo Fisher Scientific, USA).

Immunodetection of β4 was scored as absent (0), low (1), and high (2). Scorings from tissue cores belonging to the same tumor were averaged and categorized according to the following: 0–0.49 (weak), 0.5–1.49 (intermediate), 1.5–2 (high). Immunohistochemistry was performed on all slides at once, to avoid inter-assay variation. Tonsil tissue served as positive control. Simultaneously with the IHC-procedure for detecting the integrin expression, a negative tonsil control underwent the same procedure except that the primary antibodies were replaced with PBS-BSA.

### Statistical Analysis

Chi-squared tests were used to study the relation between clinicopathological and tumor characteristics and level of β4 expression. Missing values were included as missing indicator. Cox proportional hazard models with the lowest group (β4-weak) as reference were used to investigate whether there was an association between the intensity of β4 in the tumor tissue and overall or disease-free survival. Here, overall survival was defined as the period of the patient in which no death occurred during the follow-up time, which ended at the last date of follow-up (1st of January 2012). Disease-free survival was defined as the period of the patient in which no tumor or metastasis associated with colon cancer was observed during the follow-up time. Analyses were done with IBM SPSS Statistics version 25 and statistical tests were two-sided and considered significant if *p* < 0.05.

## Results

**Fig. 1:**
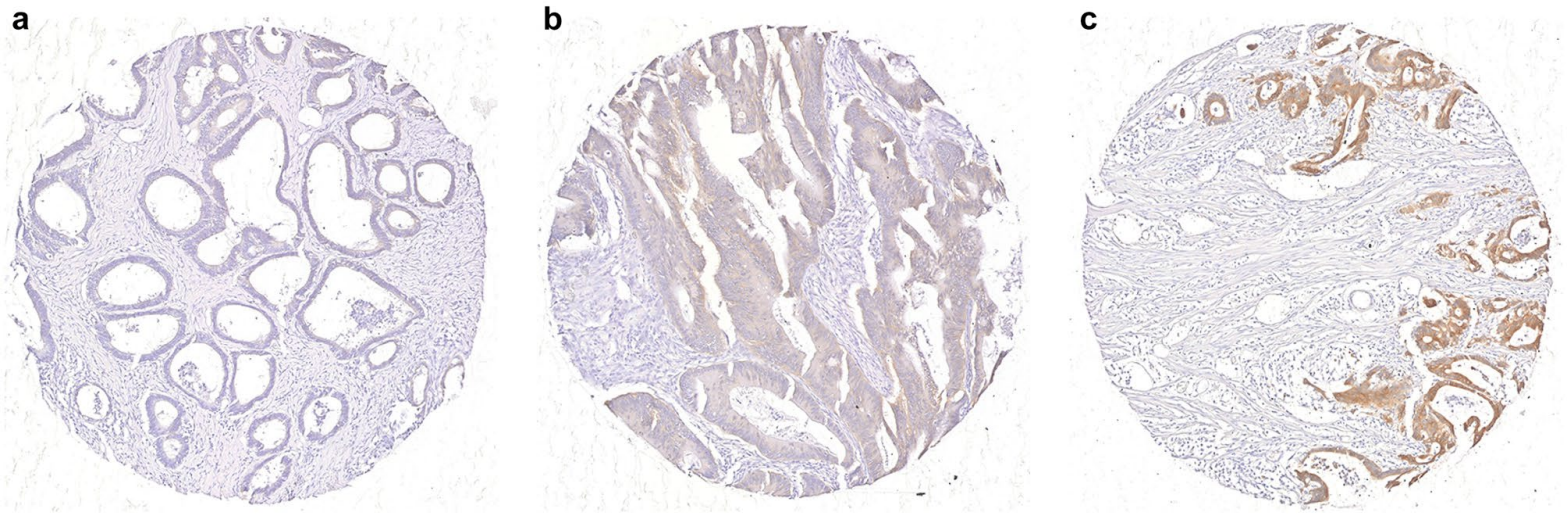
Difference in integrin β4 expression on the tumor. Integrin β4 expression in three different TMA cores, showing the difference in expression between **a** weak, **b** intermediate, and **c** high β4 expression in tumor cells (anti-integrin β4 monoclonal antibodies, × 10)

To investigate whether there was an association between survival in colon cancer patients and β4 expression in tumor tissues, the expression of this molecule was determined in 653 colon cancers. β4 expression was solely detected in cancer cells and not in stromal cells (Fig. 1a–c). According to the levels of β4 expression, tumor samples were categorized into weak (β4-weak, *n* = 132, Fig. 1a), intermediate (β4-intermediate, *n* = 404, Fig. 1b), and high expression (β4-high, *n* = 117, Fig. 1c) groups. Two patients without information on tumor stage were excluded from further analyses, resulting in a cohort of 651 patients. Descriptive characteristics of these patients according to β4 intensity can be found in Table 1.

**Table 1:** Relation between integrin β4 expression on the tumor and clinicopathological characteristics (Chi-squared tests)

	**All patients**	**β4-weak**	**β4-intermediate**	**β4-high**	***p* value**
	***N***	***N* (%)**	***N* (%)**	***N* (%)**	
**Sex**					
Female	304	53 (17.4)	197 (64.8)	54 (17.8)	
Male	347	79 (22.8)	205 (59.1)	63 (18.2)	
**Age category (years)**					
≤ 65	218	35 (16.1)	149 (68.3)	34 (15.6)	
66–74	206	49 (23.8)	115 (55.8)	42 (20.4)	
≥ 75	227	48 (21.1)	138 (60.8)	41 (18.1)	
**Location**					
Proximal	311	65 (20.9)	182 (58.5)	64 (20.6)	
Distal	327	66 (20.2)	212 (64.8)	49 (15.0)	
NOS^a^	13	1 (7.7)	8 (61.5)	4 (30.8)	
**Cancer stage**					0.138
Stage 1	91	17 (18.7)	50 (54.9)	24 (26.4)	
Stage 2	264	46 (17.4)	173 (65.5)	45 (17.0)	
Stage 3	195	48 (24.6)	119 (61.0)	28 (14.4)	
Stage 4	101	21 (20.8)	60 (59.4)	20 (19.8)	
**Pathological tumor stage**					0.012
pT1	28	5 (17.9)	16 (57.1)	7 (25.0)	
pT2	77	13 (16.9)	43 (55.8)	21 (27.3)	
pT3	418	78 (18.7)	262 (62.7)	78 (18.7)	
pT4	128	36 (28.1)	81 (63.3)	11 (8.6)	
**Metastatic status**					0.842
No metastasis	550	111 (20.2)	342 (62.2)	97 (17.6)	
Metastasis	101	21 (20.8)	60 (59.4)	20 (19.8)	
**Differentiation status**					0.097
Good differentiated	54	15 (27.8)	28 (51.9)	11 (20.4)	
Moderately differentiated	419	83 (19.8)	272 (64.9)	64 (15.3)	
Poorly differentiated	127	22 (17.3)	75 (59.1)	30 (23.6)	
**MMR status**					0.259
MMR proficient	581	117 (20.1)	361 (62.1)	103 (17.7)	
MMR deficient	54	11 (20.4)	32 (59.3)	11 (20.4)	

^a^*NOS,* not other specified

**Fig. 2:**
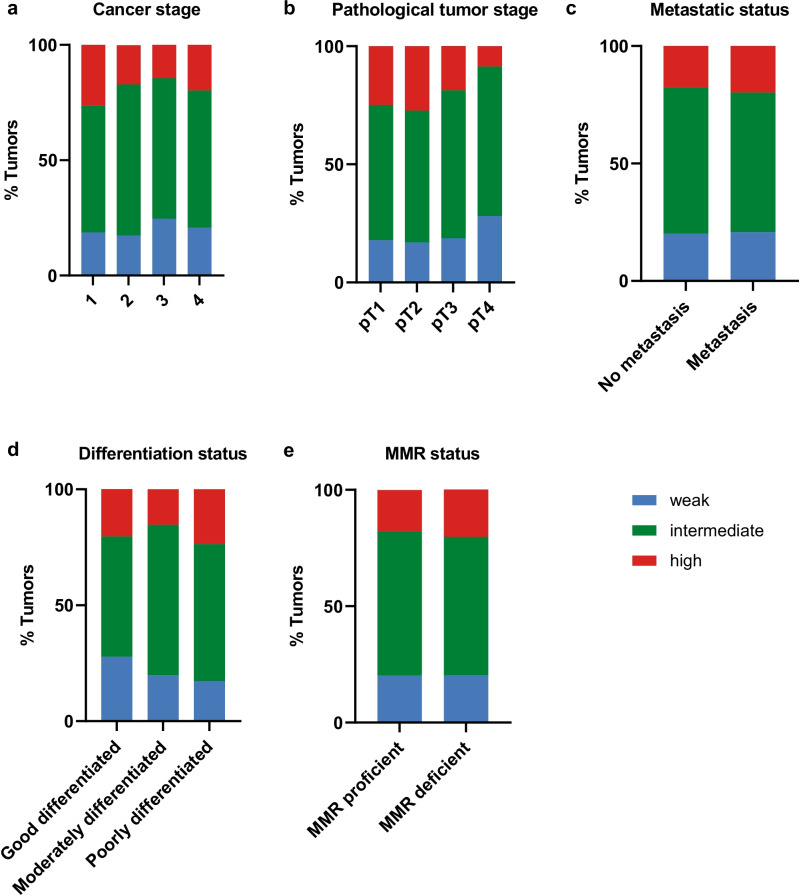
Integrin β4 expression on the tumor and relation with clinicopathological characteristics. **a** Relation between β4 expression and cancer stage (stage 1–4). **b** Relation between β4 expression and pathological tumor stage (pT1–pT4). **c** Relation between β4 expression and metastatic status (no metastasis/metastasis). **d** Relation between β4 expression and differentiation status (good/moderately/poor). **e** Relation between β4 expression and MMR status (MMR proficient/MMR deficient)

When looking into other parameters, no association could be observed between β4 expression and clinical cancer stage (*p* = 0.138, Fig. 2a). However, we did observe that at advanced pathological tumor (pT) stage, pT4, tumors generally expressed lower levels of β4 (weak expression level of 28.1% in the pT4 tumors versus 17.9% in pT1 tumors). This might suggest a relation between this integrin expression and invasion of the primary tumor (*p* = 0.012, Fig. 2b). No association was observed between β4 expression and metastatic status of the tumor (*p* = 0.842, Fig. 2c), differentiation grade (*p* = 0.097, Fig. 2d), or the mismatch repair (MMR) status of tumors (*p* = 0.878, Fig. 2e).

**Fig. 3:**
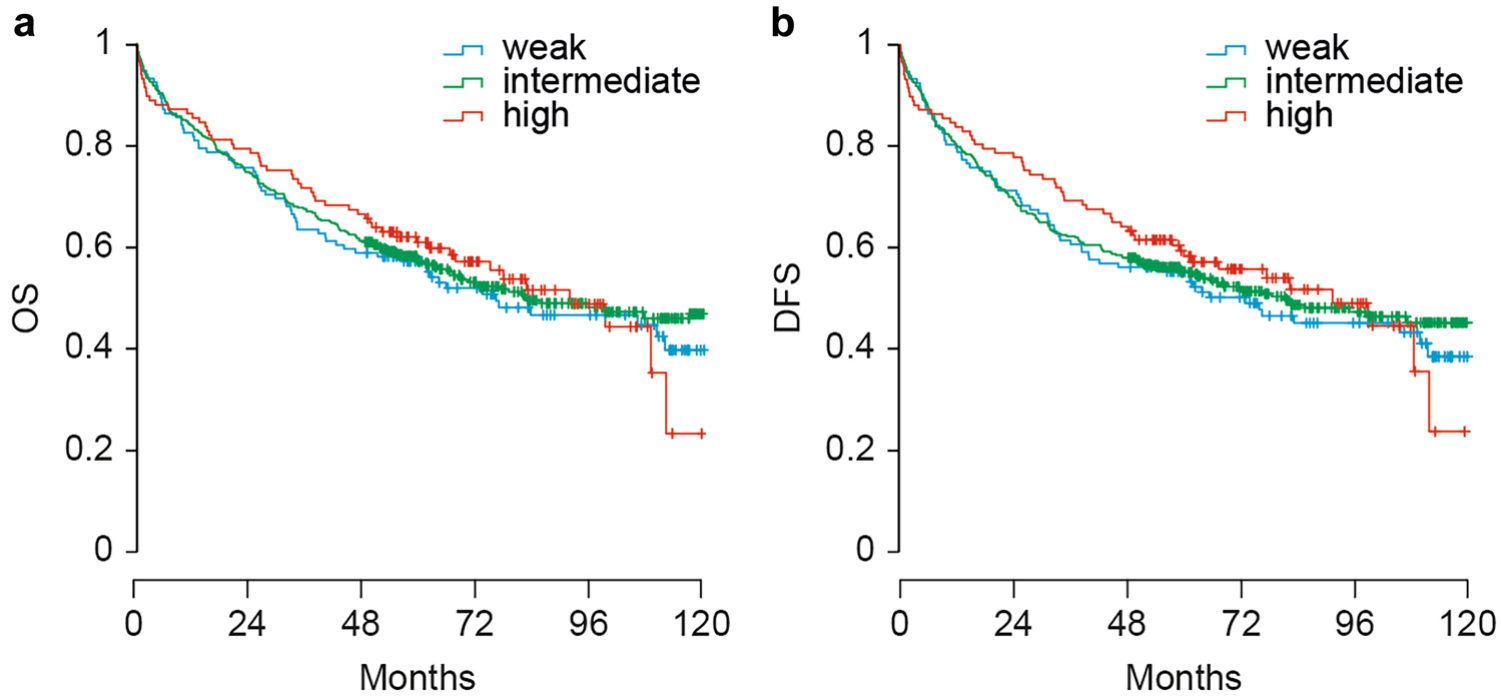
Integrin β4 expression on the tumor and relation with overall and disease-free survival. **a** Survival curve for overall survival reported in months for all groups with β4-weak used as reference group. **b** Survival curve for disease-free survival reported in months for all groups with β4-weak used as reference group

Secondly, the association between β4 expression groups and survival was investigated. When looking into the overall survival, median survival time was 6.85 years (95% CI 5.43–8.27) and there were 313 events. There were no differences in overall survival among the three groups according to β4 expression (*p* = 0.845, Fig. 3a), with an HR of 0.934 (95% CI 0.708–1.232; *p* = 0.629) for the intermediate expression as compared to weak expression and HR 0.907 (95% CI 0.635–1.294; *p* = 0.598) for the high expression as compared to weak expression. For the disease-free survival, median survival time was 6.77 years (95% CI 5.24–8.31) and there were 324 events. There were no differences in disease-free survival among the three groups according to β4 expression (*p* = 0.767, Fig. 3b), with an HR of 0.938 (95% CI 0.715–1.231; *p* = 0.647) for the intermediate expression as compared to weak expression and HR 0.878 (95% CI 0.618–1.247; *p* = 0.468) for the high expression as compared to weak expression.

To investigate whether there was a difference in β4 expression between cancer and normal tissues, we assessed the expression of this integrin in 28 samples of healthy colonic mucosa. No differences could be observed in terms of expression levels or cellular localization of this integrin (e.g., membranous, diffuse).

## Discussion

Integrins play a fundamental role in cell migration and tumor invasion. Integrin α6β4 has been proposed to be involved in such processes, namely, by contributing to hemidesmosome disassembly and PI3K and Ras signaling activation, leading to malignant characteristics like proliferation, migration, and apoptosis resistance [2, 8, 10]. Previous studies have failed to reach a consensus about a potential association between β4 expression and the aggressive phenotype invasive behavior of colorectal cancer cells [19–25]. Therefore, we sought to investigate the prognostic role of β4 expression in a large cohort of colon cancer patients.

Assessment of expression of β4 in a cohort of 651 colon cancers did not reveal any association between expression level and patient overall or disease-free survival. Additionally, no differences were observed between β4 expression level and the metastatic stage of the tumor. However, high expression of this integrin was less frequent in tumors that had penetrated through the colonic wall (pT4). Also, we did not find an association between β4 expression and tumor grade, although it was previously proposed that cellular differentiation impacted the expression of this integrin. In colorectal cancer, differentiation is tightly linked to the mismatch repair status of tumors which could constitute an important confounder when performing such analysis. However, we also demonstrated that no differences in β4 expression can be observed when comparing mismatch repair-deficient or -proficient cancers.

This is the first study looking at a large (*n* = 651) colon cancer cohort to explore the prognostic value of β4 expression. Another recently published study did look at the relation between ITGB4 gene expression level and clinical outcomes of colorectal cancer patients [25]. Despite a significant association between ITGB4 expression and an unfavorable overall survival was reported, contradictory data are presented in the same publication. Two recent studies associated β4 expression by immunohistochemistry to patient outcome. Zhang and colleagues described that higher β4 expression was associated to worse patient survival [23]. However, the authors could not discriminate differences between tumor stages. In contrast to our study, that study did demonstrate that β4 expression was significantly higher in tumor tissues when compared to normal colon, whereas we did not observe any difference between tumor and healthy colon tissue. Another study evaluated the expression of β4 in relation to tumor budding features in the tissues derived from 232 stage 2 colorectal cancer patients [28]. Tumor budding is a known poor prognostic factor in stage 2 CRC but the authors demonstrated that by incorporating information on β4 expression, the prognostic significance of tumor budding increased considerably: tumor buds that displayed higher expression of β4 were associated with worse patient prognosis. Nevertheless, the overall expression of β4 alone was not predictive for clinical outcome, in line with our study. We have not attempted to replicate these observations, as we considered that scoring of tumor budding would not be reliable on a TMA. Sordat and colleagues also described that β4 expression was found decreased in carcinomas, particularly at tumors buds, when compared to adenomatous and healthy tissue thereby arguing against a role for the overexpression of this protein in invasive and migratory processes [24]. One could also propose that loss of β4 expression would promote hemidesmosome disassembly and migratory properties of cancer cells. This could explain the observation that this protein is less frequently expressed at advanced pT stages in our cohort.

In part, the conflicting reports regarding the role of β4 in colorectal cancer may be explained by the existence of different isoforms of this protein and of its dimer partner (α6) [31, 33]. A β4 isoform that lacks its cytoplasmic domain (β4^ctd−^) was described to be expressed in normal colonic mucosa but largely absent in cancer cells [22]. Also, a distinction can be made between two α6 subunits, where the α6A isoform is generally more frequent than the α6B isoform in colon and lung tumors [33, 34]. The different possibilities of integrin heterodimer formation involving β4 could thus confound the association of β4 expression with clinical prognosis in colon tumors.

Limitations with the current study include the use of a TMA for the evaluation of β4 expression which could complicate the identification of heterogeneous patterns of expression, for instance, between tumor core and invasive front. However, this is partially compensated by the fact that each tumor was represented by three different cores distributed in separate TMAs as well as by the inclusion of a large number of samples. Secondly, different isoforms of integrin β4 were shown to exert distinct functions while the current immunohistochemical detection did not allow the discrimination of those. The same applies for the possibility that different α6 subunits can form a complex with β4.

## Conclusion

In sum, this is the largest study investigating the relation between β4 integrin expression and colon cancer-related clinicopathological outcomes and shows no association. The apparent lack of an association with overall and disease-free survival does not exclude a role for the β4 integrin, as we did find a lower expression at advanced stages of invasion which argues against the hypothesis that higher β4 levels relate to the acquisition of invasive properties by cancer cells.

## Data Availability

Data sharing is not applicable to this article as no datasets were generated or analyzed during the current study.
